# On Reduction of Dislocations by Manipulation

**Published:** 1884-07-20

**Authors:** M. M. Louis Hutchinson

**Affiliations:** Chicago


### ON REDUCTION OF DISLOCATIONS BY MANIPULATION.

By M. M. LOUIS HUTCHINSON, L.R.C.S.I., ETC., CHICAGO.

I would wish to invite your attention to the reduction of dislocations by manipulation, in the hope that others, becoming interested in the matter, might assist us by their information and experience. When we consider that it is the most scientific method of procedure, inasmuch as for its skillful and successful application it entails a knowledge of anatomy and mechanics; and that it possesses the advantages of being easily applied without distress to the patient, and with such little exertion on the part of the surgeon that he can usually dispense with any assistance, and that, moreover, in the event of its failure the other methods can be tried immediately after. Surely it is a subject worthy of more attention than it would seem to receive at present. It is seldom made the theme of a lecture, clinical or other; the text-books award it only a fleeting notice, and consequently the student devotes little study to it, as being a subject of no importance, and the average practitioner ignores it altogether, and has at once recourse to one of the forcible methods. I do not presume to disparage these methods, as I know full well that there are cases where they alone are successful; but I venture to submit that in the majority of recent simple dislocations—especially at the hip and shoulder-joints—manipulation should first get a fair trial. Occasionally, the flexions, rotations, etc., that have been described by surgeons, will not suffice, and then the surgeon must try other movements, impossible to clearly describe, but which will invariably suggest themselves to his intelligence—the manœuvres of French surgeons—and by these means he will seldom fail to obtain success.

When a student, I was first attracted by the facilities and advantages of this procedure, and in my own practice I have obtained the most satisfactory results from it. With your permission, I shall briefly describe my last case.

—— Wright, wagon-driver, aged thirty-five, suffered from a recent subcoracoid dislocation at the right shoulder-joint. He had had six previous experiences of this dislocation, it occurring with him from the slightest cause. In every case it had been reduced after considerable difficulty by the heel-in-axilla or other of these methods, and he complained that the force employed in these efforts was such that he always suffered a long time from its effects. I placed him in the dorsal recumbent position, and standing a little behind and to his right side, I placed my left hand on his shoulder, with the fingers behind and the thumb in front—this enabling me at once to steady the part and to feel and guide the head of the displaced humerus—which in all such dislocations can be distinctly felt beneath the coracoid process. I next, with my right hand, seized his arm by the wrist in the extended position, this affording me the greatest leverage, and raised it in the line of his body to an angle of about 90°; then semi-rotating outwords, I made the very slightest extension, and the bone glided at once into place. I bandaged the arm to his side, gave the usual directions, and in a fortnight the man had resumed his occupation, quite well, thus showing that a more speedy and complete recovery follows the reduction of dislocations by manipulation.—*Jour. Amer. Med. Association.*

